# Systematic evaluation of head motion on resting‐state functional connectivity MRI in the neonate

**DOI:** 10.1002/hbm.26183

**Published:** 2022-12-28

**Authors:** Jung‐Hoon Kim, Josepheen De Asis‐Cruz, Kushal Kapse, Catherine Limperopoulos

**Affiliations:** ^1^ Developing Brain Institute, Children's National Washington District of Columbia USA

**Keywords:** data censoring, fMRI, functional connectivity, head motion, neonate

## Abstract

Reliability and robustness of resting state functional connectivity MRI (rs‐fcMRI) relies, in part, on minimizing the influence of head motion on measured brain signals. The confounding effects of head motion on functional connectivity have been extensively studied in adults, but its impact on newborn brain connectivity remains unexplored. Here, using a large newborn data set consisting of 159 rs‐fcMRI scans acquired in the Developing Brain Institute at Children's National Hospital and 416 scans from The Developing Human Connectome Project (dHCP), we systematically investigated associations between head motion and rs‐fcMRI. Head motion during the scan significantly affected connectivity at sensory‐related networks and default mode networks, and at the whole brain scale; the direction of motion effects varied across the whole brain. Comparing high‐ versus low‐head motion groups suggested that head motion can impact connectivity estimates across the whole brain. Censoring of high‐motion volumes using frame‐wise displacement significantly reduced the confounding effects of head motion on neonatal rs‐fcMRI. Lastly, in the dHCP data set, we demonstrated similar persistent associations between head motion and network connectivity despite implementing a standard denoising strategy. Collectively, our results highlight the importance of using rigorous head motion correction in preprocessing neonatal rs‐fcMRI to yield reliable estimates of brain activity.

## Graphical abstract

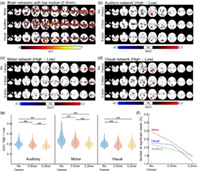

## 1 INTRODUCTION

Resting‐state functional connectivity MRI (rs‐fcMRI) provides unprecedented access to the functional organization of the brain, including neural network development (Allen et al., 2011; Biswal et al., 2010; Dosenbach et al., 2010), network pattern formation under different brain states (Gonzalez‐Castillo et al., 2015), and circuitry alterations in the setting of disease (Buckner et al., 2009; Lynall et al., 2010; Plitt et al., 2015; Zeng et al., 2012). In children and adults, resting state networks (RSNs) have proven to be consistent across subjects and repeatable within individuals (Finn et al., 2015; Hu et al., 2022). Such reliable and robust connectivity estimates, however, rely on minimizing the influence of noise, including head motion‐induced artifacts, on the measured brain signal.

Current evidence from adults suggests that head movement during scan confounds functional connectivity, such as significantly biasing the effect of between‐group difference in case–control studies (Haller & Bartsch, 2009; Power et al., 2012; Van Dijk et al., 2012), reducing the strength of functional connectivity between distant brain regions (Power et al., 2012) or within higher‐order RSNs, (e.g., default mode network, DMN) (Van Dijk et al., 2012), or introducing false activation patterns (Jolly et al., 2020; Power et al., 2012; Van Dijk et al., 2012; Yang et al., 2005). Similarly, in infants and toddlers, one study from Kaplan et al., (Kaplan et al., 2022) showed that the respiratory‐related head motion artifact confounds within‐subject reliability of functional connectivity. Thus, motion correction plays a critical role in the preprocessing of resting state images, especially in noncooperative participants such as fetuses, newborns, and young children who tend to have higher head motion (Greene et al., 2018; Power et al., 2019; Yuan et al., 2009).

In adults, prospective (Kundu et al., 2012; Spreng et al., 2019) and retrospective (Griffanti et al., 2014; Jo et al., 2010; Power et al., 2012; Pruim et al., 2015) strategies have been proposed to mitigate the impact of head motion on rs‐fcMRI. Retrospective techniques are more widely utilized as it can be applied after images have been acquired. Here, we assessed the impact of retrospective motion correction on neonatal rs‐fcMRI. Early research demonstrated that regression of translational and rotational estimates of head motion and their transformations significantly attenuated the association between motion and blood‐oxygen‐level‐dependent (BOLD) signal fluctuations (Fox et al., 2009; Friston et al., 1995); this approach is now widely used in rs‐fcMRI research. However, recent studies suggested that while helpful, nuisance regression failed to address brain‐wide systematic effects of head motion on functional connectivity, possibly leading to inaccurate BOLD estimates (Power et al., 2012; Satterthwaite et al., 2012; Van Dijk et al., 2012). To address this, censoring or scrubbing of high motion frames was utilized (Barnes et al., 2012; Jones et al., 2010; Kennedy & Courchesne, 2008; Power et al., 2012; Power et al., 2015; Smyser et al., 2010; Smyser et al., 2011).

Using censoring, volumes with excessive frame‐by‐frame head motion (i.e., frame‐wise displacement or FD) were excluded from the analysis. Frame‐wise displacement consolidates translational and rotational motion into a single value. A FD threshold of 0.5 mm is widely used, following suggestions proposed in (Power et al., 2012). Later, more stringent threshold levels were suggested, as it was shown to be more effective in suppressing the influence of head motion on rs‐fcMRI (Siegel et al., 2014; Yan et al., 2013). Specifically, it can boost the signal‐to‐noise level of fMRI data, leading to increased detection power of group‐wise effect and improved reproducibility of findings across different data sets (Marchitelli et al., 2017; Yan et al., 2013; Zeng et al., 2014; Zuo et al., 2014).

The influence of head motion on functional connectivity estimates in newborns has not been systematically studied. Likewise, the effectiveness of data censoring in neonatal rs‐fcMRI is largely unknown. To address this gap, we investigated how head motion and censoring of high motion volumes at different FD thresholds impact newborn rs‐fcMRI. A total of 575 data sets from healthy, term newborns recruited at Children's National Hospital Developing Brain Institute and a publicly available data set (*The Developing Human Connectome Project* or dHCP) were analyzed. In these independent data sets, we demonstrated the significant influence of head motion on neonatal rs‐fcMRI that persisted despite standard nuisance regression strategies. We then evaluated the efficacy of scrubbing high motion frames. We hypothesized that like in adult rs‐fcMRI, data censoring would minimize the biasing effect of head motion on neonatal rs‐fcMRI.

## 2 MATERIALS AND METHODS

### 2.1 Subjects

We acquired 159 scans from 157 healthy newborns (mean ± *SD* postmenstrual age/PMA, in weeks: 41.67 ± 1.84; PMA range: 37.71–47.73 weeks; 86 M/71F) who were recruited as controls for a longitudinal study investigating brain development in healthy and high‐risk neonates at Children's National in Washington DC. The detailed demographics of subjects can be found at Table 1. Of the 157 subjects, 10 were born before 37 weeks. Apart from 22 newborns, all were scanned during the neonatal period. Among 157 subjects, 135, 20, and 2 were appropriate, small, and large for gestational age (AGA, SGA, LGA), respectively. Here, SGA and LGA was defined as smaller than 10th percentile or larger than 90th percentile for a given GA (Duryea et al., 2014). Maternal exclusion criteria were psychiatric disorders, metabolic disorders, genetic disorders, complicated pregnancies, multiple pregnancies, alcohol, tobacco use, maternal medications, and contraindications to MRI. Exclusion criteria for the neonates were dysmorphic features by antenatal ultrasound, chromosomal abnormalities by amniocentesis, presentation after 28 gestational weeks, multiple gestation, or evidence of congenital infections. All experiments were conducted in accordance with the guidelines of the Institutional Review Board (IRB) of Children's National. Written parental informed consent was obtained from all participants of the study.

**TABLE 1:** Demographic data of subjects

Demographics (*n* = 157)	Mean ± *SD*	Range
PMA at scan (weeks)	41.67 ± 1.84	37.71–47.73
GA at birth (weeks)	39.26 ± 1.42	32.14–41.86
# of female	71	
Birth weight (g)	3288 ± 438	1600–4184
Apgar 1 (median; IQR 25|75)	8; 8|9	1–10
Apgar 5 (median; IQR 25|75)	9; 9|9	5–10

*Note*: Birth weight not available for one subject, Apgar 1 and 5 not available for eight subjects.

Abbreviations: GA, gestational age; *SD*, standard deviation; PMA, post‐menstrual age.

### 2.2 MRI acquisition

We used a 3T scanner (Discovery MR750, GE Healthcare, Milwaukee, WI) to acquire structural and functional MR images. Scanning parameters were as follows: T2‐weighted fast spin‐echo anatomical images, TR = 2500 ms; TE = 64.49 ms, voxel size = 0.625 × 1 × 0.625 mm; resting state images, TR = 2000 ms, TE = 35 ms, voxel size = 3.125 × 3.125 × 3 mm, flip angle = 60 degrees, field of view = 200 mm, and matrix size = 64 × 64. Prior to the scan, babies were fed, swaddled in a warm blanket, immobilized using an infant vacuum pillow, and equipped with ear plugs/earmuffs. They were scanned asleep and unsedated. Heart rate and oxygen saturation were monitored by a nurse during the scan. A total of 200 volumes (=6.7 min) were collected from all participants except for two neonates who had 280 (=9.3 min) and 150 (=5 min) volumes each.

### 2.3 Preprocessing of neonate rsfMRI data

Functional MR images were preprocessed using AFNI, ANTS, and in‐house MATLAB code (Avants et al., 2009; Cox, 1996; De Asis‐Cruz et al., 2018). First, we performed slice‐wise head motion correction to fix within volume slice misalignment. Next, we corrected slice‐timing effects, discarded the first four volumes of each data set to stabilize the magnetization, de‐spiked the time series, and applied bias‐field correction. Resting state images were then motion‐corrected by registering EPI data to a base volume, co‐registered to the T2 image, and normalized to a standard neonate brain template using linear and nonlinear transformations. All three steps, plus resampling to a 3‐mm isotropic grid, were performed at once to avoid repeated resampling of the data. The motion correction step produced the 6‐parameter motion estimates (three rotational parameters: yaw, pitch, roll; three translational parameters: *x*‐, *y*‐, *z*‐axis) used for computing frame‐by‐frame motion (described later). We then performed intensity normalization (Ojemann et al., 1997) and smoothed the data using a 5 mm full‐width half‐maximum (FWHM) kernel. Lastly, bandpass filtering (0.009–0.08 Hz) and nuisance regression were simultaneously performed (Hallquist et al., 2013). For the regression, the nuisance variables included in the study were (1) six motion estimates, their first‐derivates and quadratic from the rigid‐body analysis of head motion (Friston et al., 1996); (2) signals averaged over localized white matter (Jo et al., 2010); and (3) first three principal components of the ventricular CSF signal (Behzadi et al., 2007; Muschelli et al., 2014). Lastly, we evaluated the effect of global signal regression (GSR) on motion‐related variance in the scans (Figure 6). Global signal was defined as the average signal across timeseries within gray matter voxels; the calculated global signal was regressed out from the voxel‐wise timeseries. For each scan, brain voxels were automatically labeled into one of nine tissue labels (cerebrospinal fluid, cortical gray matter, white matter, nonexistent, lateral ventricles, cerebellum, deep gray, brainstem, and hippocampus) using a previously validated deep learning segmentation algorithm (Zhao et al., 2022). Once completed, we visually inspected the quality of the tissue segmentation and manually corrected the images, if necessary. Volumes where >10% of voxels were classified as intensity outliers were excluded in the analysis. Qualitative assessment of the images after preprocessing revealed suboptimal co‐registration in seven subjects. Those were excluded from the analysis yielding a total of 149 scans from 148 neonates (83 M/65F) in the final data set.

### 2.4 Measurement of frame‐wise displacement

Per frame, translational (*x*‐, *y*‐, and *z*‐axis) and rotational (roll, pitch, and yaw) head motion parameters were obtained from the motion correction step described above. After converting rotational parameters defined in radians to millimeter scale by modeling the brain as a sphere with radius of 30 mm (60% of adult brain originally designed in (Power et al., 2012)), FD at *i‐*th timepoint was computed by measuring the displacement of the head relative to the previous position and averaging the six parameters as ${\mathrm{FD}}_{i}=\frac{\left|∆d_{\mathrm{ix}}\right|+\left|∆d_{\mathrm{iy}}\right|+\left|∆d_{\mathrm{iz}}\right|+\left|∆\phi_{i}\right|+\left|∆\theta_{i}\right|+\left|∆\psi_{i}\right|}{6}$, where $d_{\mathrm{ix}}$, $d_{\mathrm{iy}}$, and $d_{\mathrm{iz}}$ stand for positional displacement at *x*‐, *y*‐, and *z*‐axis, respectively, and $∆\phi_{i}$, $∆\theta_{i}$, and $∆\psi_{i}$ stand for roll, pitch, and yaw, respectively. $∆d_{\mathrm{ix}}=d_{\left(i-1\right)x}-d_{\mathrm{ix}}$, likewise for other parameters, $d_{\mathrm{iy}}$, $d_{\mathrm{iz}}$, $∆\phi_{i}$, $∆\theta_{i}$, and $∆\psi_{i}$. ${\mathrm{FD}}_{0}$ was set to 0. For each data set, volume‐to‐volume FD were averaged for the full scan to obtain the subject's average motion or FD.

### 2.5 Data censoring

We evaluated the effects of high motion on functional connectivity by censoring (“scrubbing”) brain volumes at two different FD thresholds—0.5 and 0.2 mm—based on thresholds established in studies in adults (Power et al., 2012; Siegel et al., 2014; Yan et al., 2013). A more stringent cut‐off would expectedly yield a shorter time series. Thus, to prevent the possible confounding effect of different signal‐to‐noise levels due to differences in residual volumes after censoring (Nebel et al., 2022), all data sets were capped at 4 min scan length. For each censored data, mean FD was measured by averaging FD over survived volumes.

### 2.6 Temporal signal‐to‐noise ratio

We assessed rs‐fcMRI data quality by computing the temporal signal‐to‐noise ratio (tSNR) (Tabelow et al., 2009; Triantafyllou et al., 2005; Welvaert & Rosseel, 2013) on the bias‐field corrected volumes; temporal SNR was used as a proxy for data quality as this measure is known to be sensitivity of fMRI activation (Krüger et al., 2001; Krüger & Glover, 2001; Parrish et al., 2000) and statistical detection power (Wald, 2012). Here, tSNR at the *i‐*th voxel was defined as

$${\text{tSNR}}_{i}=\frac{{\bar{S}}_{i}}{\sigma_{i}},$$

where ${\bar{S}}_{i}$ and $\sigma_{i}$ are the mean and standard deviation, respectively, of the BOLD signal over timeseries at the *i‐*th voxel. TSNR measure was averaged across all voxels within the brain mask to yield the representative signal quality of an individual's fMRI data.

### 2.7 Functional connectivity

We furthermore evaluated the influence of motion on functional connectivity strength and pattern in sensory‐related and higher‐order cognitive networks. Specifically, we investigated functional connectivity (FC)‐FD associations at the motor, visual, auditory, and default mode networks, networks that have previously been described in newborns (Doria et al., 2010). We used the following regions as seeds: precentral, calcarine, superior temporal, and posterior cingulate. These were defined in the neonatal automated anatomical labeling (AAL) atlas composed of 90 cortical and subcortical regions of interest (ROIs) (Shi et al., 2011). Distance between ROIs was defined as Euclidean distance between centers of ROIs. For the sensory‐related networks, we evaluated both cross‐hemispheric and whole brain connectivity; for the DMN, we only assessed the latter.

For each subject, we averaged BOLD signals for voxels comprising each brain region and computed the Fisher z‐transformed Pearson's correlation between BOLD time courses from homotopic regions. Then, for the entire cohort (=149 data sets), we computed the influence of motion on network connectivity by correlating (i.e., Pearson) subjects' degree of head motion (i.e., average FD) with their left–right connectivity (i.e., cross‐hemispheric FC). We also computed the FC for every pair of ROIs (=4005 = 90 × [90–1]/2) and evaluated the influence of motion at the whole connectome level.

Next, we examined the effect on whole brain connectivity of sensory‐related networks by head motion by contrasting statistical parametric maps of low‐ versus high‐motion groups. Each subgroup comprised 25% (~37 out of 149 data sets) of the scans. The 25% with the least head motion, or lowest average FD, were placed in the low‐motion group, and vice versa for the higher‐motion group. For each scan, using seed‐based correlation with the right ROIs as seeds, we estimated a connectivity strength map reflecting the correlation between BOLD fluctuations at the seed region and BOLD fluctuations over the whole brain. Individual Pearson correlations maps were transformed into Fisher's *z*‐score maps and then averaged per group. A similar whole‐brain approach was performed to evaluate the DMN using the bilateral posterior cingulate cortices as a seed region.

The significance of within‐group connectivity patterns was tested using one‐sample *t* test at FDR‐adjusted *p* < .05; the significance of between‐group differences was tested using two‐sample *t* test (high motion > low motion; uncorrected *p* < .01 and FDR‐corrected *p* < .05). Note that groups with low/high motion were defined for each censoring condition. The size of the group‐level effect of head motion was evaluated by counting the number of voxels that were significantly different between high‐ versus low‐motion groups. We further evaluated the strength of group‐level effect of head motion by averaging the between‐group difference in network strength for all significant voxels. The significance of between‐group strength difference was tested using one‐way ANOVA, followed by post hoc two‐sample *t* test.

### 2.8 Reproducibility analysis using developing human connectome project database

To ensure that the motion effects on neonatal fcMRI observed in our data set were not biased by our preprocessing choices nor properties inherent to our data set, we performed a similar analysis on a large, publicly available newborn rs‐fcMRI data set (*n* = 416 neonates; mean ± *SD* postmenstrual age/PMA: 40.6 ± 2.5 weeks; PMA range: 29.3–45.1 weeks; 228 M/188 F) downloaded from http://www.developingconnectome.org. This data set was already preprocessed using the dHCP pipeline (Fitzgibbon et al., 2020). No additional preprocessing steps were applied after we downloaded the images from the database except for warping the standard brain atlas provided by dHCP to the neonatal AAL brain template. Each dHCP rs‐fcMRI scan was composed of 2300 volumes (~15 min); spatial resolution was 2.15 mm isotropic and TR = 0.392 s. Acquisition parameters for the dHCP data set can be found at (Fitzgibbon et al., 2020). One major preprocessing difference in the dHCP pipeline, compared to our DBI data set, was their use of automated independent component analysis‐based denoising technique, ICA‐FIX (Griffanti et al., 2014; Salimi‐Khorshidi et al., 2014). Briefly, ICA‐FIX auto‐identifies ICA components carrying artifact/noise by comparing these, so called, “bad” components, to the manually identified component set. Once identified, clean data was extracted from noisy fMRI data by excluding bad components. For additional details, see (Fitzgibbon et al., 2020). We also used the FD values provided by dHCP (e.g., “framewise_displacement” variable stored in “sub‐SUBID_ses‐SESSID_motion.tsv” file). The mean FD per scan was computed by averaging FD over the full time series.

After normalizing the dHCP data to our AAL template, we calculated cross‐hemispheric connectivity strength at low‐level sensory brain networks (motor, auditory and visual) using the same analysis described above. We hypothesized that there may still be residual effects of head motion in the dHCP data post ICA‐FIX, analogous to the residual FC‐motion associations observed in adults after standard motion regression strategies. Our approach to evaluate the influence of motion on network connectivity in the dHCP data set differed from our previously described approach for the Children's National data set. As previous work using the dHCP data set showed that connectivity at the sensory regions (auditory, visual, and sensorimotor) increased with PMA (Eyre et al., 2021), we investigated that the age‐dependency of network strength could be further enhanced by incorporating head motion in the association model. As the first step, like the previous, the relationship between motion and connectivity was quantified. We evaluated the bias of head motion in the dHCP data set, by comparing the degree of association between PMA and network measures with‐ and without controlling the effect of head motion. The association between PMA and different network measures was estimated by simple correlation analysis or partial correlational analysis by controlling the effect from head motion. To test the statistical significance of difference in association between PMA and network strength, a Bootstrapping resampling method was applied. Specifically, the association between PMA and network strength were measured using a subset (=50%) of samples that were randomly selected from the whole scans, and the whole procedure was repeated 5000 times, each with a random selection of subset.

## 3 RESULTS

### 3.1 Associations between FD and PMA

**FIGURE 1:**
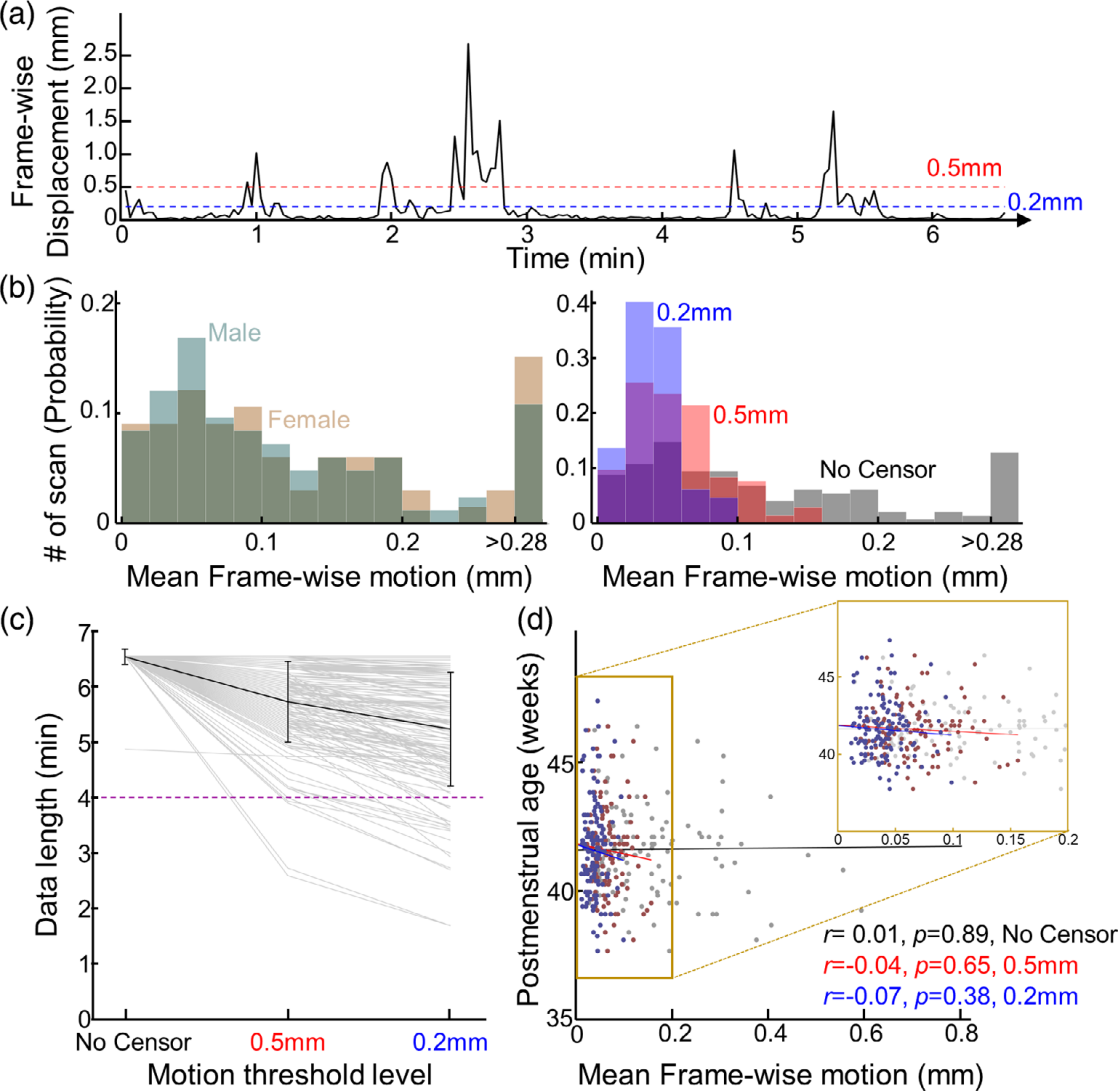
Frame‐wise displacement (FD) in newborns. (a) Representative motion trace, FD over time, in one subject. (b) (Left) Mean head motion distribution without censoring was similar for males (green) and females (brown). (Right) Probability histogram of mean FD in neonates (*n* = 149). (c) Maximum brain volumes available decrease at stricter thresholds: without scrubbing (=120 vols) > 0.5 mm >0.2 mm; light gray line = scan volumes per subject, black line = mean data length, error bar = standard deviation. Purple dashed line indicates length of analyzed data (d) FD was not correlated with PMA; black line = line of best fit; inset zooms in on scrubbed data

We explored the distribution of head motion in the neonatal cohort (n = 149). Figure 1a shows a representative single‐subject motion (FD) trace. There were long periods of low motion interspersed with sudden surges of head motion (Figure 1a). When high‐motion volumes were not scrubbed, the maximum mean FD among scans was 0.85 mm (Figure 1b; Table 2, mean ± *SD*; 0.13 ± 0.13 mm), higher than reported values in normative adults (Mayer et al., 2007; Seto et al., 2001; Van Dijk et al., 2012; Yan et al., 2013). When high motion volumes were censored at 0.5 and 0.2 mm, average FD decreased to 0.06 ± 0.03 mm and 0.04 ± 0.02 mm, respectively. Unlike in adults (Van Dijk et al., 2012) or children (Yuan et al., 2009), head motion in males (0.13 ± 0.14 mm) and females (0.14 ± 0.11 mm) was not statistically different (Figure 1b inset; two‐sample *t* test; *p* = .54). Scrubbing data at different thresholds reduced the number of volumes available for further analyses. As expected, the number of censored volumes increased with stricter thresholds: 12.3% ± 11.0% (maximum: 26.0%) at 0.5 and 20.8% ± 15.7% (maximum: 37.6%) at 0.2 (Figure 1c). Related to this, the number of scans with data length <4 min was 3/ and 17/149 scans at 0.5 and 0.2 mm, respectively. Head motion was not correlated with PMA (Figure 1d, no scrubbing: *r* = 0.01, *p* = .98; 0.5 mm: *r* = −0.04, *p* = .65; 0.2 mm: *r* = −0.07, *p* = .38).

**TABLE 2:** Distribution of frame‐wise displacement at different threshold levels

Threshold level	Min‐max FD (mm)	Mean FD (mm)	Median FD (mm)	SD of FD (mm)
No censor	0.01–0.85	0.13	0.10	0.13
0.5 mm	0.01–0.16	0.06	0.05	0.03
0.2 mm	0.01–0.10	0.04	0.04	0.02

Abbreviations: FD, frame‐wise displacement; *SD*, standard deviation.

### 3.2 Effect of head motion on temporal signal to noise ratio level of fMRI data

**FIGURE 2:**
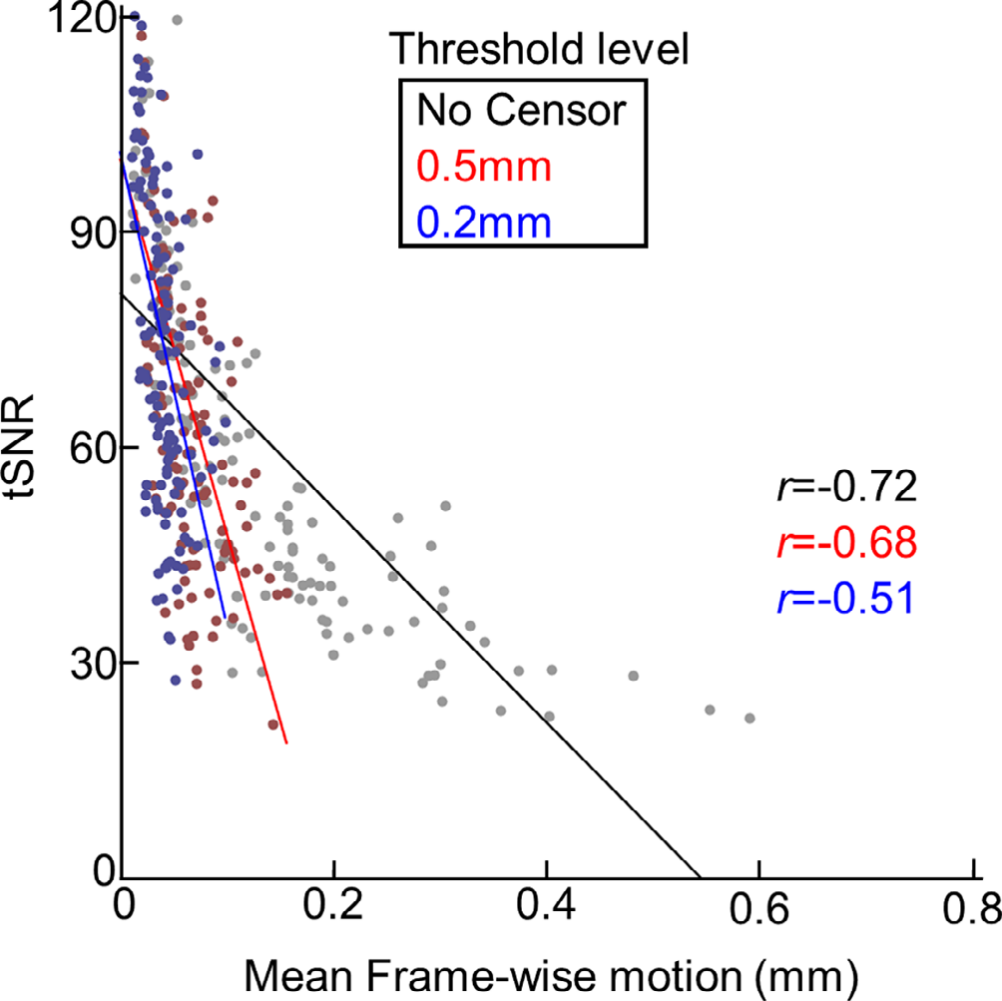
TSNR decreases with increases in FD (*n* = 149) lines represent best fit at each threshold. All *r* scores are significant, *p* < 10^−4^

Like adults (Van Dijk et al., 2012), higher head motion in newborns was associated with lower tSNR (Figure 2). Data censoring effectively reduced the association between degree of head motion and TSNR (*r* = −0.72 for no censoring, *r* = −0.68 for 0.5 mm, and *r* = −0.51 for 0.2 mm), however, the significant negative relationship persisted (*p* < 10^−4^ for all cases).

### 3.3 Association of head motion to the FC strength

**FIGURE 3:**
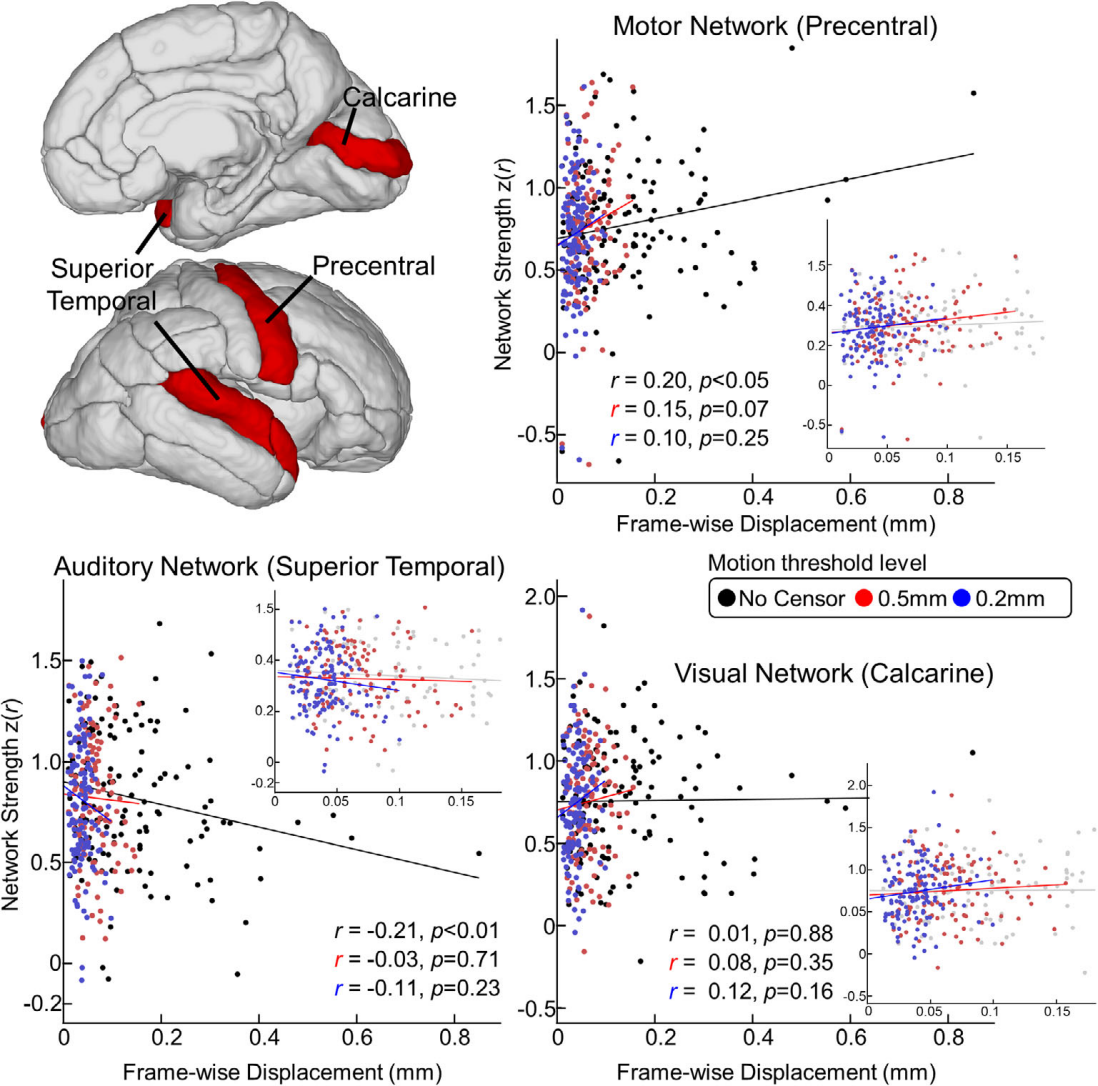
Motion is related to FC strength in motor and auditory areas. (left top) Shows sensorimotor ROIs. Scatterplots show relationship between mean head motion (FD) and connectivity strength in motor, auditory, and visual networks in 149 data sets. The lines stand for the best linear fit. Inset scatterplot is the zoomed‐in version for scrubbed data. Different colors represent different censoring threshold levels (black: no censor; red: 0.5 mm; blue: 0.2 mm)

We then explored the relationship of the degree of head motion and cross‐hemispheric connectivity at sensorimotor regions that are components of the visual, auditory, and motor networks (Figure 3, left top). The direction of association between FD and FC showed regional dependence (Figure 3). FD significantly correlated with FC in auditory and motor regions; however, censoring eliminated FD‐FC association. Interestingly, the functional coupling between bilateral motor regions increased with FD (Figure 3, right top; *r* = 0.20, *p* < .05); the reverse was observed for the auditory network (Figure 3, left bottom; *r* = −0.21, *p* < .01). FD was not correlated to the cross‐hemispheric connectivity strength at the calcarine region (Figure 3, right bottom; *r* = 0.01, *p* = .88). Data censoring at a moderate motion threshold level (=0.5 mm) eliminated the association between head motion and network strengths (0.5 mm; motor, *r* = 0.15, *p* = .07; auditory, *r* = −0.03, *p* = .71). Data censoring at 0.2 mm diminished the significant FD‐FC relationship for motor, auditory, and visual networks; it was also the most effective in reducing the correlation between head motion and motor network FC (*r*‐value = 0.20 vs. 0.15 vs. 0.10 for no censor, 0.5 mm, 0.2 mm, respectively). When scans with excessive head motion (average FD >0.5 mm) were excluded in the analysis, instead of censoring strategy (Figure S1), we found that the similar result in the motor network (*r* = 0.12, *p* = .15) but not effective for the auditory network (*r* = −0.21, *p* < .05). Lastly, the influence of head motion on FC remained largely consistent when limiting scans of interest to the AGA and full‐term babies (*n* = 117; Figure S2). Together, these results demonstrate the significant confounding effect of head motion on neonatal rs‐fcMRI and the regional dependence of the direction of FC alterations. More importantly, our results showed that data censoring can effectively minimize the effects of head motion.

**FIGURE 4:**
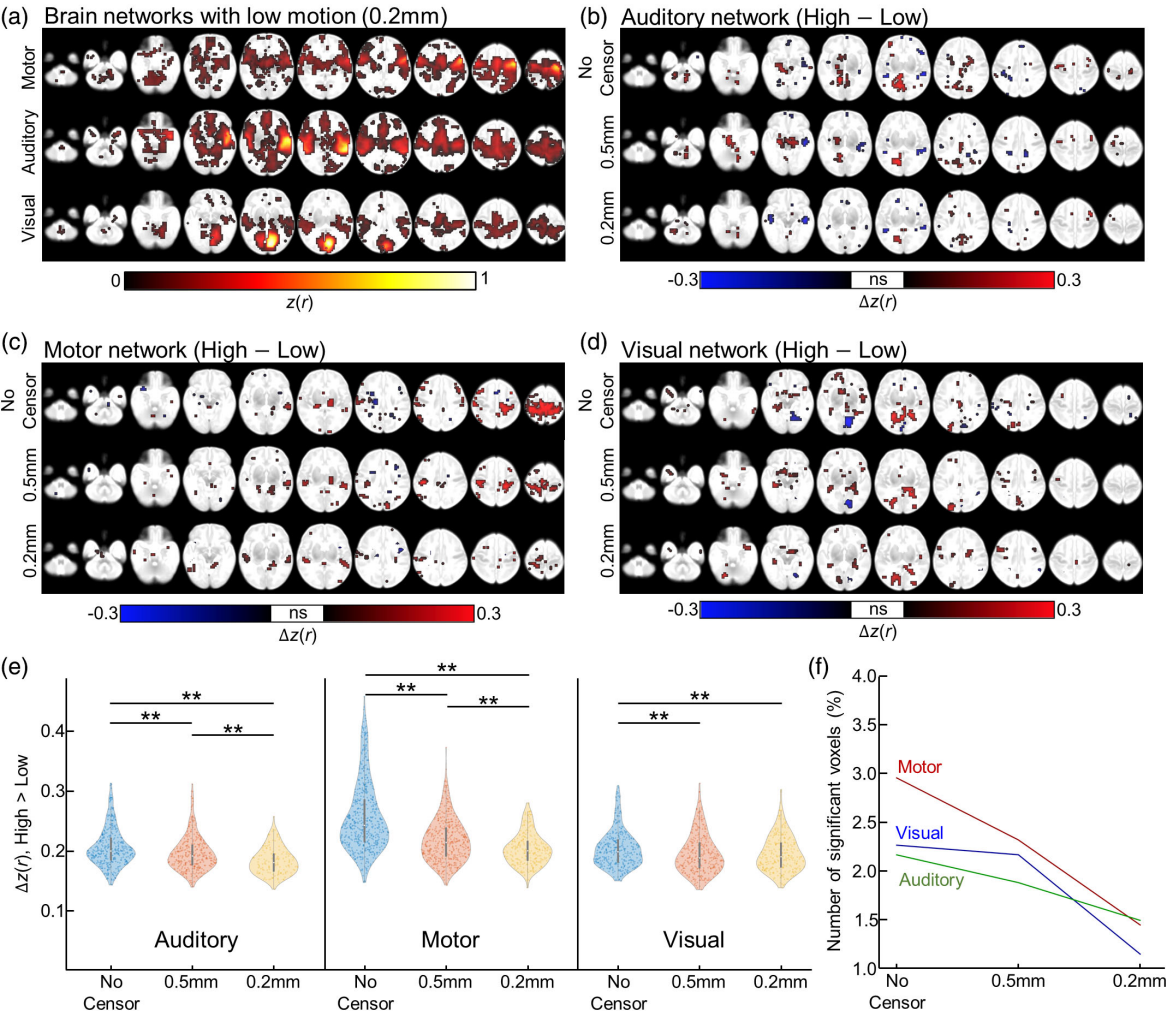
The influence of head motion on large‐scale sensory‐related brain networks. (a) Motor, auditory, and visual networks estimated by seed‐based correlation analysis. The seed regions of motor, auditory, and visual networks were the precentral, superior temporal, and calcarine cortices, respectively. (b–d) The difference in the network patterns (b: auditory network; c: motor network; d: visual network) between higher motion group versus lower motion group with various censoring strategies (top: no censor, middle: 0.5 mm, and bottom: 0.2 mm). (e) The distribution of functional connectivity strength differences between higher‐ and lower motion groups in auditory, motor, and visual networks, that is, △*z*(*r*) = network FC strength in high motion group—network FC strength in low motion group. Each dot in the violin plot represents a voxel where strength in higher motion group > lower motion group. (F) The number of voxels with significant group difference. **p‐value <.01

Data censoring furthermore effectively improved connectivity estimates at sensory‐related brain networks (Figure 4). At the 0.2 mm threshold, we identified bilateral activation at the precentral region, superior temporal region, and calcarine region, corresponding to motor, auditory, and visual networks, respectively (Figure 4a). By comparing activation maps from higher‐ and lower‐motion groups, we assessed the head‐motion‐induced blurring effects on various sensory‐related brain networks (Figure 4b,d). For all sensorimotor‐related brain networks, we observed that the data censoring strategy reduced the blurring pattern due to head motion, where the size of the blurring positively scaled with the size of the threshold, such that least blurring was observed at the most stringent motion threshold level (=0.2 mm; bottom panel for Figure 4b–d). At both threshold levels (0.5 and 0.2 mm), between‐group differences, ∆ *z*(*r*); FC map averaged over high‐motion group minus the map averaged over low‐motion group, were significantly suppressed compared to no censoring (Figure 4e). Finally, censoring at the most stringent threshold (=0.2 mm) showed the least number of voxels confounded by head motion (1.4%, 1.1%, and 1.5% for motor, auditory, and visual networks, respectively), compared to uncensored data or data with motion threshold = 0.5 mm (Figure 4f; 0.5 mm, 2.3%, 2.2%, and 1.9%; no censor, 2.9%, 2.3%, and 2.2%; motor, auditory, and visual networks, respectively). When combined with a stricter FDR threshold (FDR‐corrected *p* < .05), data censoring at 0.2 mm yielded no voxels confounded by head motion, while uncensored data still showed voxels with significant FD‐FC relationship (Figure S4; uncensored data: 0.82%, 0.005%, and 0.12%; motor, auditory, and visual networks, respectively). This result may suggest that better sensitivity of findings can be achieved by combining preprocessing design (e.g., data censoring) and analytical design (e.g., stricter statistical threshold), rather than by a single design.

**FIGURE 5:**
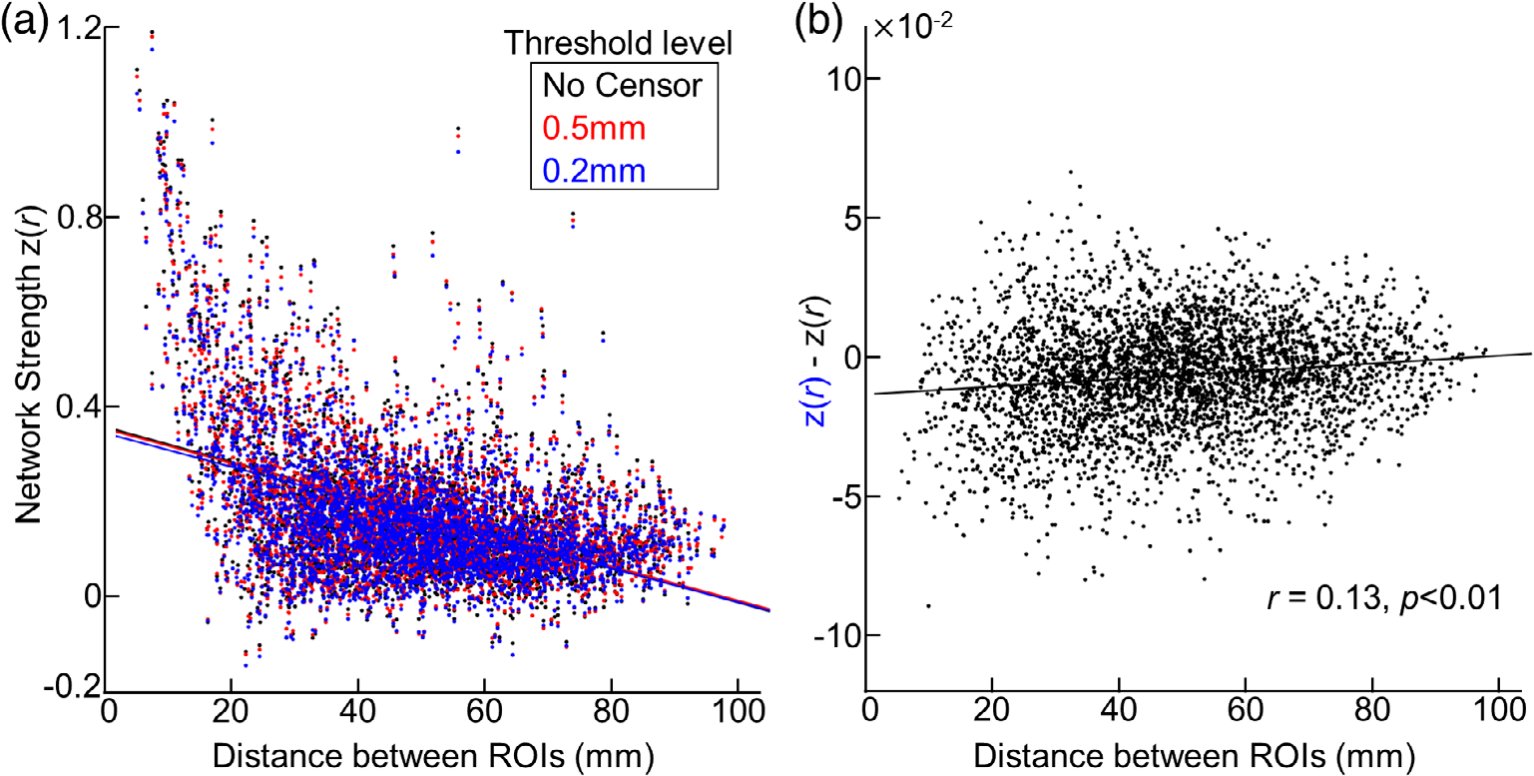
The influence of newborn head motion on whole brain functional connectivity. (a) The scatter plot between distance between ROIs and inter‐ROI connectivity strength. The connectivity strength is estimated by averaging over scans. Each dot stands for the FC between a pair of 90 ROIs. *n* = 4005. (b) The difference between network strength estimated from data without censoring and one from censored data (threshold =0.2 mm). The calculated difference is plotted as a function of distance between ROIs.

**FIGURE 6:**
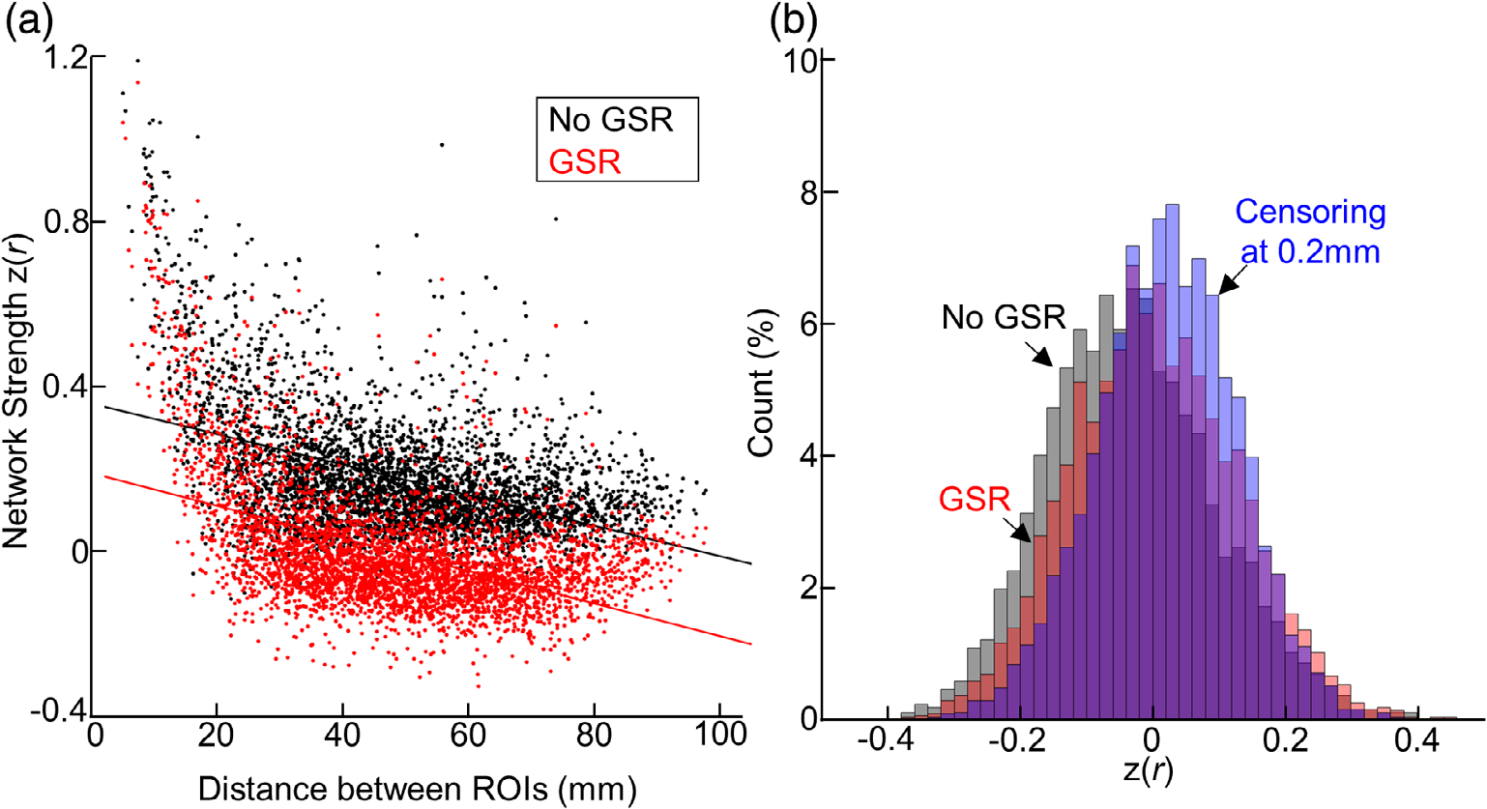
The impact of global signal regression (GSR) on the influence of head motion. (a) Scatter plot between inter‐ROI distance and inter‐ROI connectivity strength. The connectivity strength is estimated by averaging over 149 scans. Each dot stands for the connectivity between a pair of 90 ROIs. *n* = 4005. (b) The distribution of correlation between head motion and network strength of the FC between a pair of 90 ROIs.

We further extended our analysis to the whole newborn connectome. In line with observations in human adults (Power et al., 2012), we also observed that FC strength between ROIs was negatively associated with the distance between ROIs (Figure 5a; *r* = −0.45, *p* < 10^−4^). Such trends remained largely consistent, regardless of inclusion of censorship procedure. Despite of small changes in FC strength related to data censoring, we found that data censoring significantly decreased the strength of short‐distance FCs but augmented the strength of long‐distance FCs (Figure 5b; *r* = 0.13, *p* < .01). More importantly, with uncensored data, the percentage of connections significantly contaminated by motion was 6.47% (=256/4005), while the percentage decreased when lenient data censoring was applied (3.62%). With a more stringent data censoring strategy, only a few voxels had FCs (0.23%) biased by FD (Figure S3). Lastly, we investigated the effectiveness of GSR on removing the association between FD and FC (Figure 6). As shown in Figure 6a, GSR effectively set the overall strength of FCs to 0 (mean and STD of *z* = 0.00 ± 0.15), compared to the FCs without GSR (mean and STD of *z* = 0.18 ± 0.15). GSR was also effective in decreasing the number of FCs biased by FD (=4.84% vs. 6.47% without GSR), but less than data censoring strategy at 0.5 mm (=3.62%) or at 0.2 mm (=0.22%; Figure 6b). Altogether, our results suggest the head motion may bias neonatal functional connectivity patterns, but proper data censoring could effectively attenuate the confounding effect of head motion.

### 3.4 The influence of head motion on large‐scale connectivity of higher‐order functional networks

**FIGURE 7:**
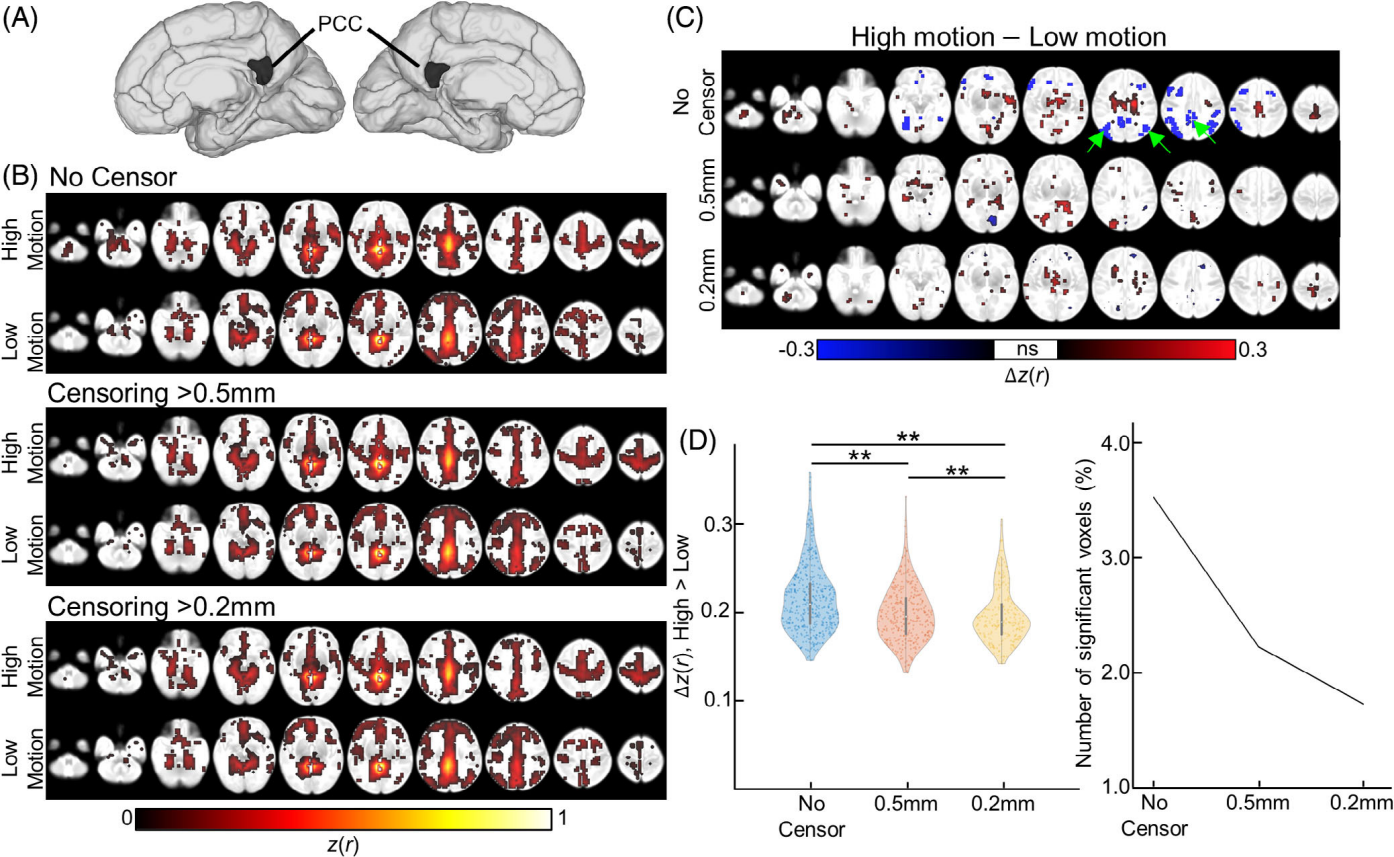
The default mode network (DMN) at different degrees of head motion. (a) Posterior cingulate cortex (PCC) was chosen as the seed ROI. (b) DMN statistical parametric maps estimated at different FD thresholds for high (top panel) and low motion groups (bottom panel). (c) High > low comparison (top: no censor, middle: 0.5 mm, and bottom: 0.2 mm). Green arrow stands for the brain regions belonging to the default mode network. (d) The distribution of differences in network strength between higher and lower motion groups, estimated using seed‐based analysis (left). Each dot stands each voxel within graymatter. The number of voxels with significant between‐group difference (right). **p‐value <.01

We examined the effects of head motion on the DMN, a higher‐order functional network. Using bilateral posterior cingulate cortices (PCC) as seeds (Figure 7a), we estimated a statistical parametric map per subject and averaged individual maps for low motion and high motion groups. The network pattern of the low motion group (~25% of 149 = 37 scans; head motion, mean ± *SD* = 0.03 ± 0.01 mm) revealed significant associations to the bilateral prefrontal regions and bilateral inferior parietal lobule (one‐sample *t* test; FDR‐corrected *p* < .05; bottom panel in Figure 7b). These areas are components of the default mode network (Raichle, 2015). Similar activations were observed in the high motion group (=37 scans; head motion, mean ± *SD* = 0.30 ± 0.14 mm) but with reduced spatial specificity of findings, or blurring, especially around the seed regions (top panel in Figure 7b). Note that there was no age difference between lower‐ and higher‐motion groups (PMA in weeks, mean ± *SD*, lower = 41.73 ± 2.04; higher = 41.49 ± 2.05; two‐sample *t* test, *p* = .62). By contrasting activations for higher‐ versus lower‐motion groups at different FD thresholds, we observed that the blurring effect due to the difference in head motion was reduced by the data censoring strategy (bottom panel in Figure 7c). Quantitatively, the size and strength of blurring pattern was suppressed by data censoring technique (Figure 7d). Specifically, after censoring the data at 0.5 and 0.2 mm, the percentage of voxels with significant between‐group difference decreased from 3.5% (no censor) to 2.2% (0.5 mm) and 1.7% (0.2 mm). Under stricter statistical threshold (FDR‐corrected *p* < .05), data censoring with the stringent threshold (=0.2 mm) yielded nearly no voxels confounded by head motion (0.005%) compared to uncensored data (Figure S4; 0.7%). In addition to the reduced size, the data censoring technique also significantly suppressed the strength of the bleeding effect, that is, the difference in network strength between low versus high motion (one‐way ANOVA; *F*[2,1650] = 51.07, *p* < 10^−4^). Followed by post hoc two‐sample *t* test, data censored at the level of 0.2 mm (∆ *z*‐score = 0.18 ± 0.03) significantly reduced network strength differences compared to one without data censoring (0.20 ± 0.04; *p* < 10^−4^) or less stringent censoring threshold = 0.5 mm (0.19 ± 0.03; *p* < .05). We also demonstrated that the network strength at the inferior parietal lobule, which is a core DMN region (highlighted as green arrow in Figure 7c), was weaker when the scan suffered from higher head motion, compared to the group with lower head motion (Figure 7c top panel; two‐sample *t* test; uncorrected *p* < .01). Conversely, head‐motion‐induced change in connectivity strength was suppressed with the data censoring strategy (Figure 7c middle and bottom panel).

### 3.5 Investigating influence of head motion on dHCP data set

**FIGURE 8:**
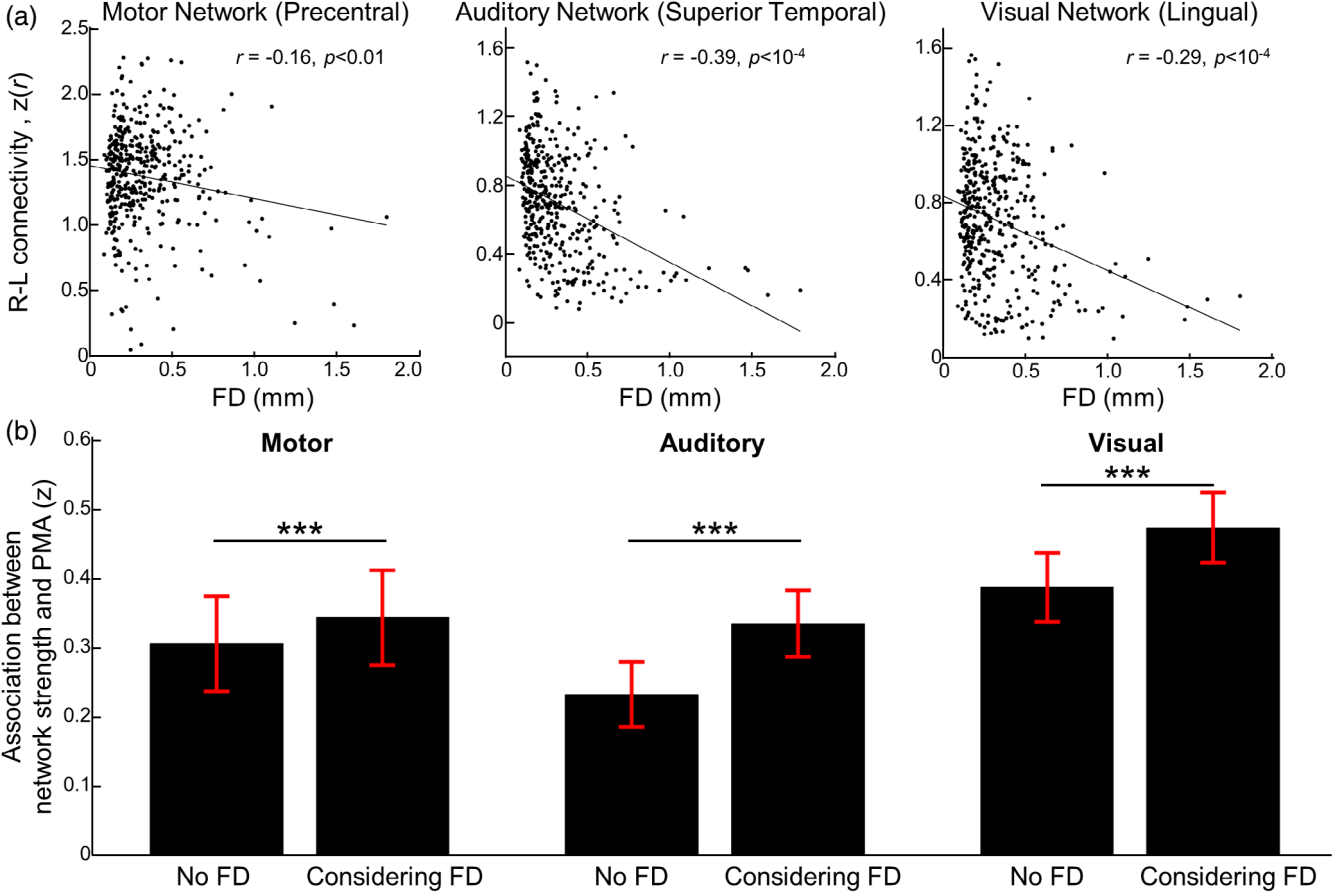
The influence of remaining head motion after ICA‐FIX data pruning step on dHCP data set. (a) Scatterplot between mean frame‐wise displacement (FD) and the bilateral connectivity strength of motor (left), auditory (middle), and visual networks (right). The linear lines stand for the best linear fit. The seed regions of motor‐, auditory‐, and visual networks were precentral region, superior temporal cortex, and lingual region, respectively. (b) Bar plot showing (left) Fisher's *z*‐transformed correlation coefficient between postmenstrual age (PMA) and network strength versus (right) Fisher's *z*‐transformed partial correlation coefficient between PMA and network strength when controlling the effect from FD. Error bar stands for the standard deviation of Fisher's *z*‐transformed correlation coefficient or partial correlation coefficient using bootstrapping resampling method. ***p‐value <10‐4

In the previous analyses, we showed that head motion affected functional connectivity between homotopic regions; more importantly, censoring minimized associations between motion and connectivity. To confirm our findings, we performed a similar analysis on newborn data from the Developing Human Connectome Project (dHCP). The dHCP scans were acquired and processed differently from our data sets. Using preprocessed dHCP rs‐fcMRI data, we measured the strength of connectivity at motor, auditory, and visual networks using the same seeds as the previous analysis. Despite data denoising using ICA‐FIX, we found significant, negative correlations between connectivity strength of brain networks and FD (Figure 8a). Compared to the motor network (*r* = −0.16, *p* < .01), auditory (*r* = −0.39, *p* < 10^−4^) and visual (*r* = −0.29, *p* < 10^−4^) networks were more confounded by the head motion. For auditory and visual networks, the network strength of scans with excessive head motion (mean FD >0.5 mm) was close to 0, but this is not the case for the motor network. Together, our results strongly suggest an important confounding effect of head motion on newborn rs‐fcMRI; an effect that persisted despite correcting for motion using commonly used strategies.

One study using dHCP data demonstrated increasing functional connectivity strength with advancing PMA (Eyre et al., 2021). In line with this, we tested whether strengthening of FC at motor, visual, and auditory networks with advancing PMA was biased by newborn head motion. Consistent with past findings, we found that connectivity strength was positively correlated with PMA (mean ± *SD* of Fisher's *z*‐transformed *r*‐value; motor, 0.31 ± 0.07; auditory, 0.23 ± 0.05; visual, 0.39 ± 0.05; *p*‐value <10^−6^ for all networks; one‐sample *t* test with bootstrapping resampling method). Interestingly, as shown in Figure 8b, the age‐dependency of FC strength was significantly enhanced when the frame‐by‐frame motion was included in the linear regression model: partial correlation coefficient ρ(PMA, network strength, head motion). Specifically, the degree of association was strengthened by a mean margin of 0.03, 0.10, and 0.08, respectively (Fisher's *z*‐transformed ρ‐value; motor, 0.34 ± 0.07; auditory, 0.34 ± 0.05; visual, 0.47 ± 0.05) and the increment was significant for all brain network (paired *t* test; *p*‐value <10^−5^ for all networks). Collectively, our results suggest that the effect of head motion is significant in the dHCP data set even though dHCP data were processed using ICA‐FIX.

## 4 DISCUSSION

Using 575 newborn rsfMRI data collected from two independent data sets, we systematically investigated the influence of head motion on neonatal functional connectivity and the effectiveness of data censoring to reduce the effect of head motion on rs‐fcMRI. For the first time, we demonstrated the influence of head motion on connectivity estimates within the sensorimotor and higher‐order cognitive networks in neonates. This confounding effect of head motion was minimized by censoring volumes with high motion (Figure 3). Stricter thresholds led to greater dissociation between motion, and network connectivity strength and pattern (Figures 3, 4, 5 and 7). We also showed that higher head motion correlated with decreased temporal SNR; this association was reduced by scrubbing high motion volumes (Figure 2). Despite rigorous denoising, persistent effects of motion on sensorimotor connectivity were also observed in the dHCP data (Figure 8), validating observations in our (Children's National Hospital) data set. Collectively, the results presented here highlight the effect of head motion on neonatal fcMRI studies and the benefits of augmenting head motion correction strategies with data censoring to further minimize the effects of motion.

We report for the first time that the influence of head motion on neonatal rs‐fcMRI persisted after the implementation of standard motion correction procedures such as regression of motion parameters and ICA‐FIX. In our data set, scrubbing high motion volumes at 0.5 and 0.2 mm significantly attenuated the effect of motion on connectivity (Figures 3, 4, 5 and 7). Moreover, in most cases, using more stringent threshold led to improved motion correction. This lines up with findings in adults, where studies showed reduced motion effects on network connectivity with volume censoring (Marchitelli et al., 2017; Power et al., 2012; Siegel et al., 2014). Interestingly, motion affected brain networks differently—weakening and strengthening network connectivity in the auditory and motor networks, respectively, and not affecting visual network connectivity (Figure 3). Such variations in the direction of FC‐FD correlations were also observed at the whole connectome level (Figure 5 and Figure S3). Similar network‐specific effects of motion have previously been reported in adults (Power et al., 2012; Siegel et al., 2014; Van Dijk et al., 2012), but the reasons for these are unclear. Previous studies speculated an interaction between motion dynamics (i.e., higher translational motion along the anterior–posterior and inferior–superior axis and rotational motion along the left–right axis; Mayer et al., 2007; Power et al., 2012; Zaitsev et al., 2017) and brain geometry (e.g., position of ROIs, Jezzard, 2012; Power et al., 2012; Yan et al., 2013). In addition to the above factors mediating neonatal rs‐fcMRI, different maturation rates over different brain networks may play an important role in neonatal rs‐fcMRI. Further investigations that are beyond the scope of this article are needed to evaluate this hypothesis. Related to variability of head motion effects, the direction and extent of the effects of head motion on visual and motor network connectivity in the two data sets varied (DBI; Figure 3; dHCP; Figure 8). This could be due to several factors including, but not limited to, different acquisition parameters, preprocessing strategies, and PMA range. Note that in both data sets, while the direction of the impact of head motion on some networks differed, the significant FC‐FD association was reduced by the motion correction strategy.

In adults, several strategies have been proposed to deal with the confounding effects of head motion on rs‐fcMRI. To name a few, (1) nuisance regression (Friston et al., 1996; Hallquist et al., 2013); (2) real‐time, prospective motion correction (Maclaren et al., 2013); (3) volume censoring (Power et al., 2012); (4) ICA‐based regression strategies (Pruim et al., 2015; Salimi‐Khorshidi et al., 2014); and (5) deep learning (Yang et al., 2020; Zhao et al., 2020). Evidence in adults supports combining motion correction techniques (Yan et al., 2013). A similar tactic is often used in newborn studies, but direct evidence supporting the efficacy of multiple techniques has not been previously demonstrated. Here, we provide a rationale for the use of censoring high motion volumes alongside regression. As previously mentioned, our results suggest that using a stringent threshold, 0.2 mm in our case, more effectively minimized the effects of head motion (Figures 3, 4, 5, 7), but at the cost of losing more data (Figure 1). Given these findings, for newborns, we recommend routine inclusion of volume censoring in the preprocessing of rs‐fcMRI data. While using a lower motion threshold would likely give better correction, we believe that the threshold level can be flexible depending on the goal of studies. For example, a reasonable reduction of motion effects was still achieved at 0.5 mm. Thus, if one wants to maximize the statistical power of a study, 0.5 mm may be a more appropriate threshold, as more data may be salvaged and/or more subjects can be included. Conversely, if superior spatial specificity is needed, a more stringent threshold such as 0.2 mm would be a better choice. In both cases, especially when a more lenient motion threshold is used, careful interpretation of results is needed. One critical caveat of data censoring is the loss of data continuity. This could limit the types of analyses applied to the data, e.g., auto‐regression (Garg et al., 2011), phase synchrony (Pedersen et al., 2018; Weaver et al., 2016; Zhang et al., 2019), or dynamic FC analysis (Jiang et al., 2022; Rashid et al., 2014). Data censoring also brings up the issue of consecutive frame requirements for resting state studies. Although this is beyond the scope of our current work, further investigations that specifically address whether the number of consecutive frames impacts neonatal rs‐fcMRI are needed to improve our understanding of FC‐FD associations. However, it is important to note that FC measured by correlation, which is a widely used technique in rs‐fcMRI studies of human adults, appears to be robust against disruptions of the temporal structure of the data (Fair et al., 2007; Van Dijk et al., 2010).

Our study has several limitations. First, as this study was retrospective, we were unable to identify factors contributing to variations in effect of head motion to neonatal rs‐fcMRI. For example, motion effects on the motor network differed between the two data sets, DBI or dHCP. Multiple factors may be at play—for example, different recording parameters (e.g., difference in TR; 2 s in DBI vs. 0.4 s in dHCP), different preprocessing steps, and different demographic characteristics, but delineating relative contributions of such factors was not feasible in the current study. Like in adults (Bianciardi et al., 2009), prospective studies, ideally with simultaneous physiological recordings, will be needed to improve our understanding of the influence of head motion in neonatal rs‐fcMRI studies. Second, since the scope of our study was to validate the influence of head motion on neonatal rs‐fcMRI even after applying the standard preprocessing step, we did not extensively survey other head motion correction techniques. Thus, it is possible that there are better strategies, e.g., multiecho imaging techniques (Power et al., 2018), wavelet despiking (Patel et al., 2014), frequency filtering (Satterthwaite et al., 2013), or deep learning (Yang et al., 2020; Zhao et al., 2020), for attenuating the influence of head motion on rs‐fcMRI data than the data censoring method used in this study. However, it is also unlikely that there is a “perfect” technique that can fully eliminate head motion artifacts on rs‐fcMRI without sacrificing signal quality, as suggested in (Yan et al., 2013). In fact, ICA‐FIX was known to be highly effective in reducing the head motion artifact (Maziero et al., 2020). However, as shown by the findings from this study (Figure 8), the dHCP data set, which was corrected by ICA‐FIX, showed persistent FD‐FC correlations. Note that, as mentioned in the dHCP study, their preprocessing steps were intentionally minimal so that the data sets could be preprocessed further (if needed) based on the study questions. Researchers should take this into consideration when using the dHCP pipeline and data sets.

## 5 CONCLUSION

In this study, we systematically demonstrated that head motion significantly influenced functional connectivity MRI in neonates. The confounding effects of head motion displayed network specificity. In our data set, with increased head motion there was increased functional coupling in the motor network; an opposite effect was observed in the auditory network. We demonstrated that data censoring was significantly effective in attenuating the impact of head motion on newborn rs‐fcMRI. Nevertheless, careful interpretation of newborn rs‐fcMRI is necessary as regression and censoring only reduce but do not eliminate the effects of head motion.

## AUTHOR CONTRIBUTIONS

Jung‐Hoon Kim, Josepheen De Asis‐Cruz, Catherine Limperopoulos: Conceptualization, investigation, visualization, writing—review and editing. Jung‐Hoon Kim, Josepheen De Asis‐Cruz, Kushal Kapse, Catherine Limperopoulos: methodology. Josepheen De Asis‐Cruz, Catherine Limperopoulos: Supervision. Jung‐Hoon Kim, Josepheen De Asis‐Cruz: Writing—original draft.

## CONFLICT OF INTEREST

The authors declare no conflict of interest.

## Supporting information


**Figure S1. Relationship between motion and FC strength in scans with average FD <0.5 mm.** Scatterplots show the relationship between mean head motion (FD) and connectivity strength in motor, auditory, and visual networks; n = 146 scans; black lines, best linear fit.
**Figure S2. Relationship between motion and FC strength in full‐term newborns with appropriate for gestational age (AGA) birth weights.** Scatterplots show the relationship between mean head motion (FD) and connectivity strength in motor, auditory, and visual networks; n = 117 scans; black lines, best linear fit.
**Figure S3.** The distribution of correlation between head motion and network strength of the FC between a pair of 90 ROIs.
**Figure S4.** Group comparison between high‐ and low motion, for various networks. The significance level was set to FDR‐corrected *q* < 0.05.

## Data Availability

The data and code that support the findings of this study are available from the corresponding author, CL, upon reasonable request.
