# Supplementary material for: Systematic evaluation of head motion on resting‐state functional connectivity MRI in the neonate
Source: Hum Brain Mapp. 2022 Dec 28;44(5):1934–48. doi: 10.1002/hbm.26183 (PMC9980896; doi:10.1002/hbm.26183)
Supplement: Supplementary file 1 — Figure S1. Relationship between motion and FC strength in scans with average FD <0.5 mm. Scatterplots show the relationship between mean head motion (FD) and connectivity strength in motor, auditory, and visual networks; n = 146 scans; black lines, best linear fit. Figure S2. Relationship between motion and FC strength in full‐term newborns with appropriate for gestational age (AGA) birth weights. Scatterplots show the relationship between mean head motion (FD) and connectivity strength in motor, auditory, and visual networks; n = 117 scans; black lines, best linear fit. Figure S3. The distribution of correlation between head motion and network strength of the FC between a pair of 90 ROIs. Figure S4. Group comparison between high‐ and low motion, for various networks. The significance level was set to FDR‐corrected q < 0.05. [file HBM-44-1934-s001.docx]

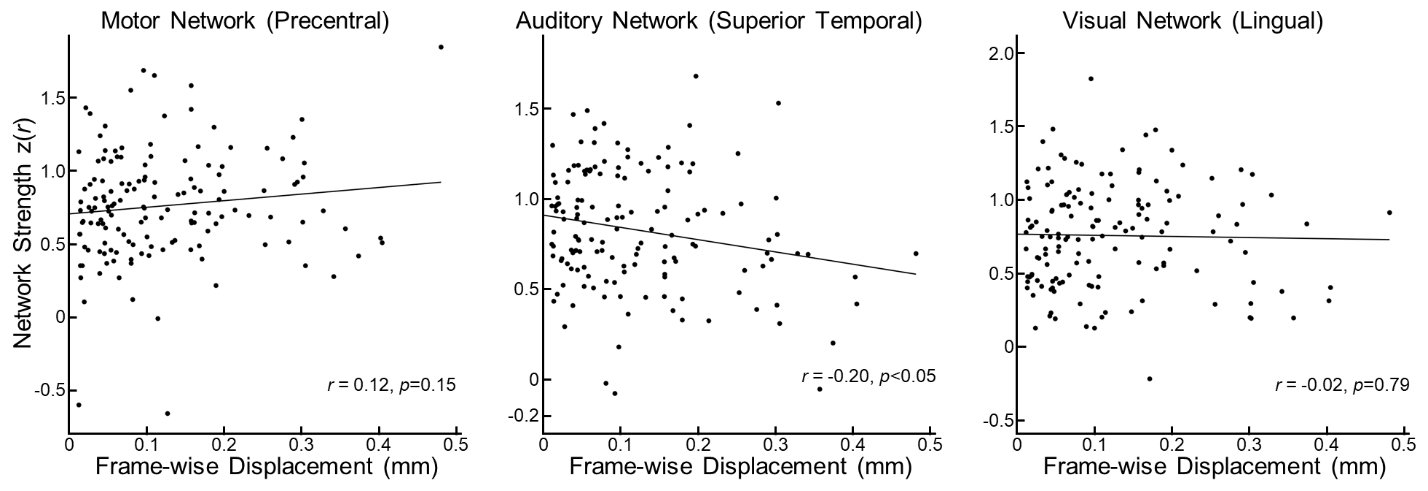


**Supplementary Figure 1. Relationship between motion and FC strength in scans with average FD < 0.5mm.** Scatterplots show the relationship between mean head motion (FD) and connectivity strength in motor, auditory, and visual networks; n=146 scans; black lines, best linear fit.


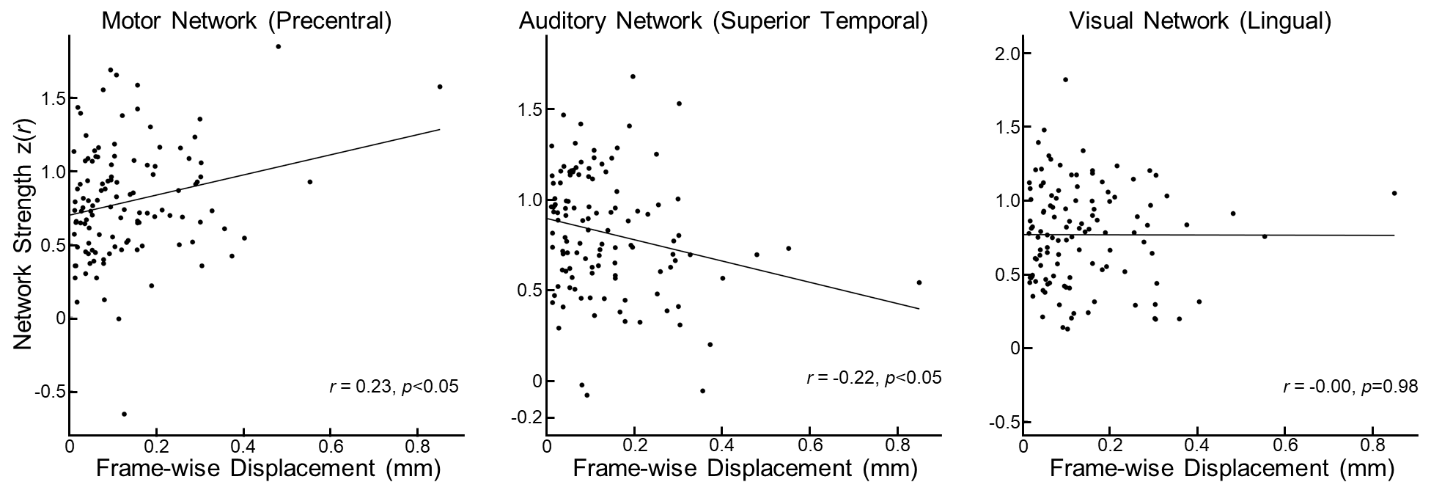


**Supplementary Figure 2. Relationship between motion and FC strength in full-term newborns with appropriate for gestational age (AGA) birth weights.** Scatterplots show the relationship between mean head motion (FD) and connectivity strength in motor, auditory, and visual networks; n = 117 scans; black lines, best linear fit.


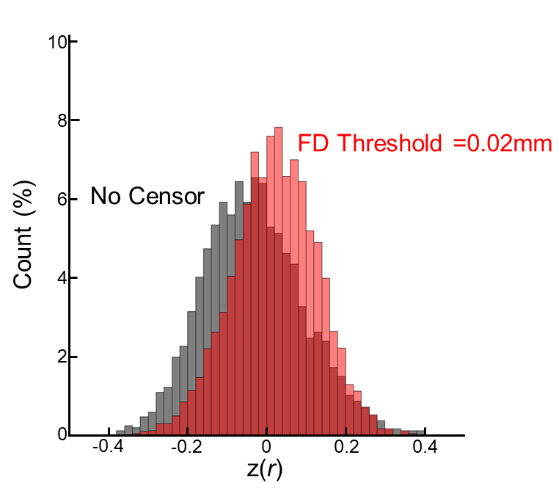


**Supplementary Figure 3.** The distribution of correlation between head motion and network strength of the FC between a pair of 90 ROIs.


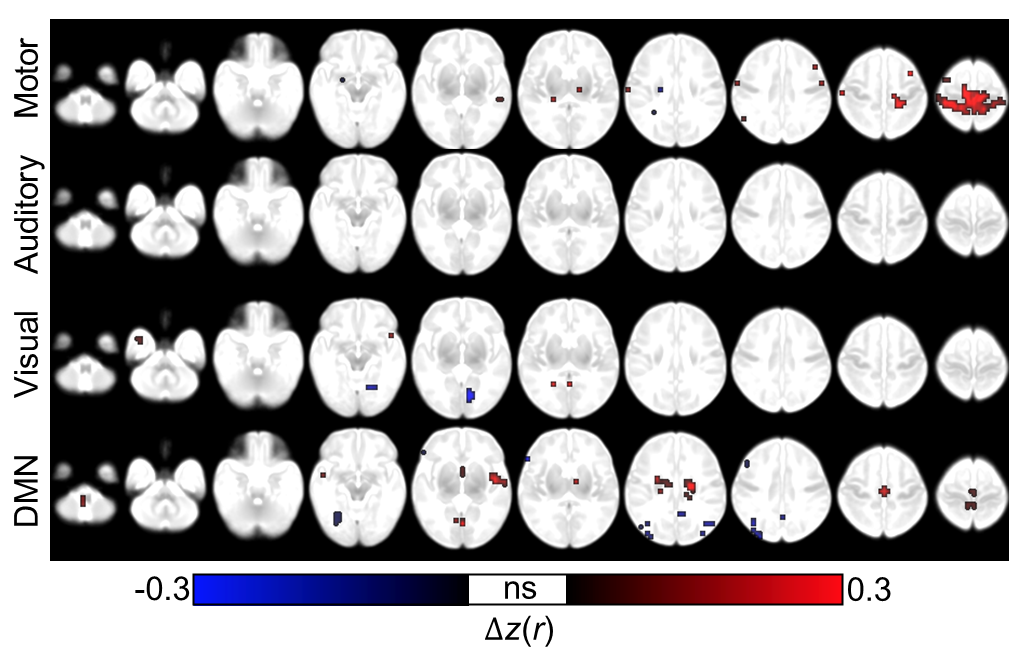


**Supplementary Figure 4.** Group comparison between high- and low motion, for various networks. The significance level was set to FDR-corrected *q*<0.05.
